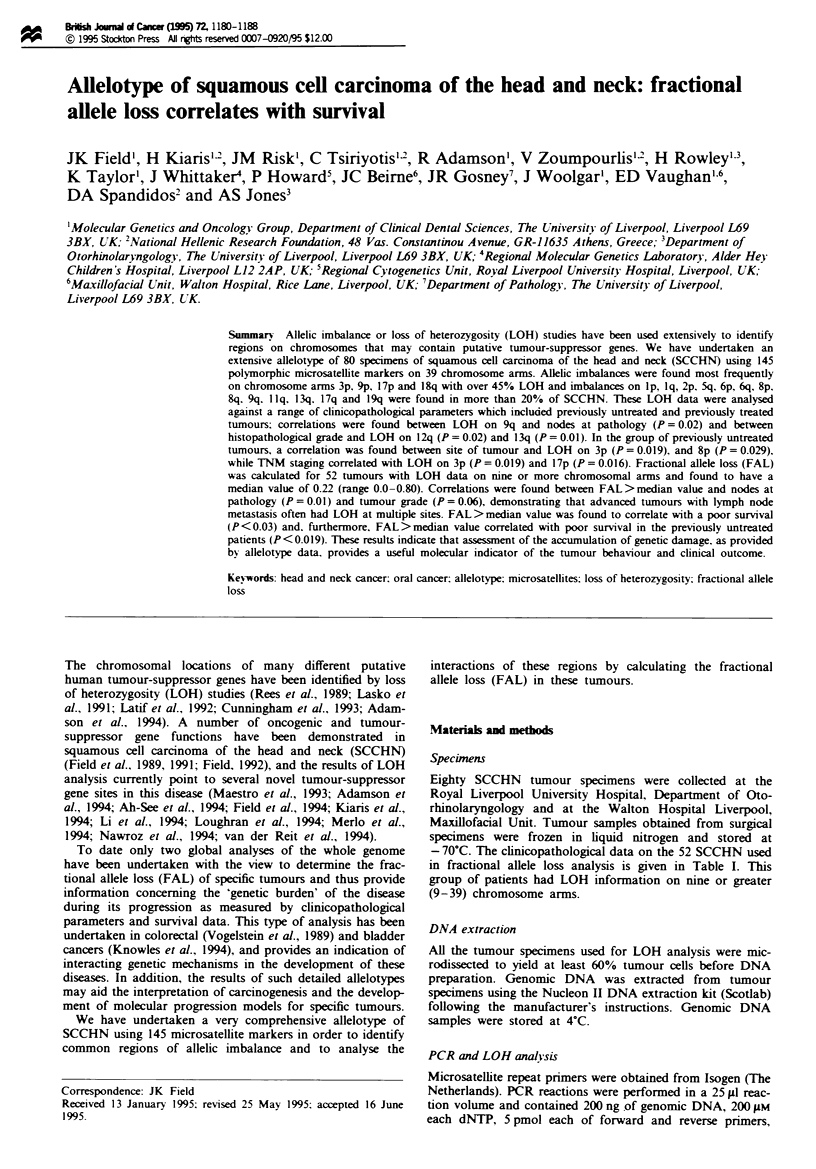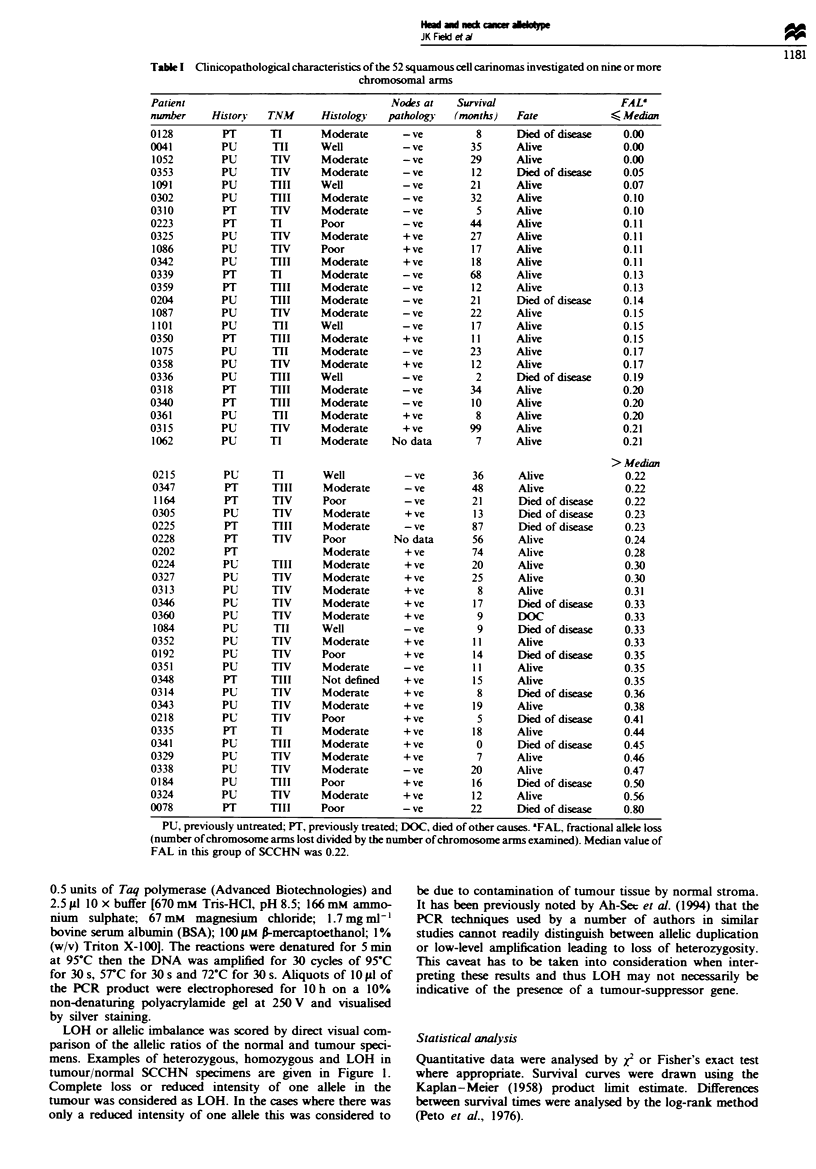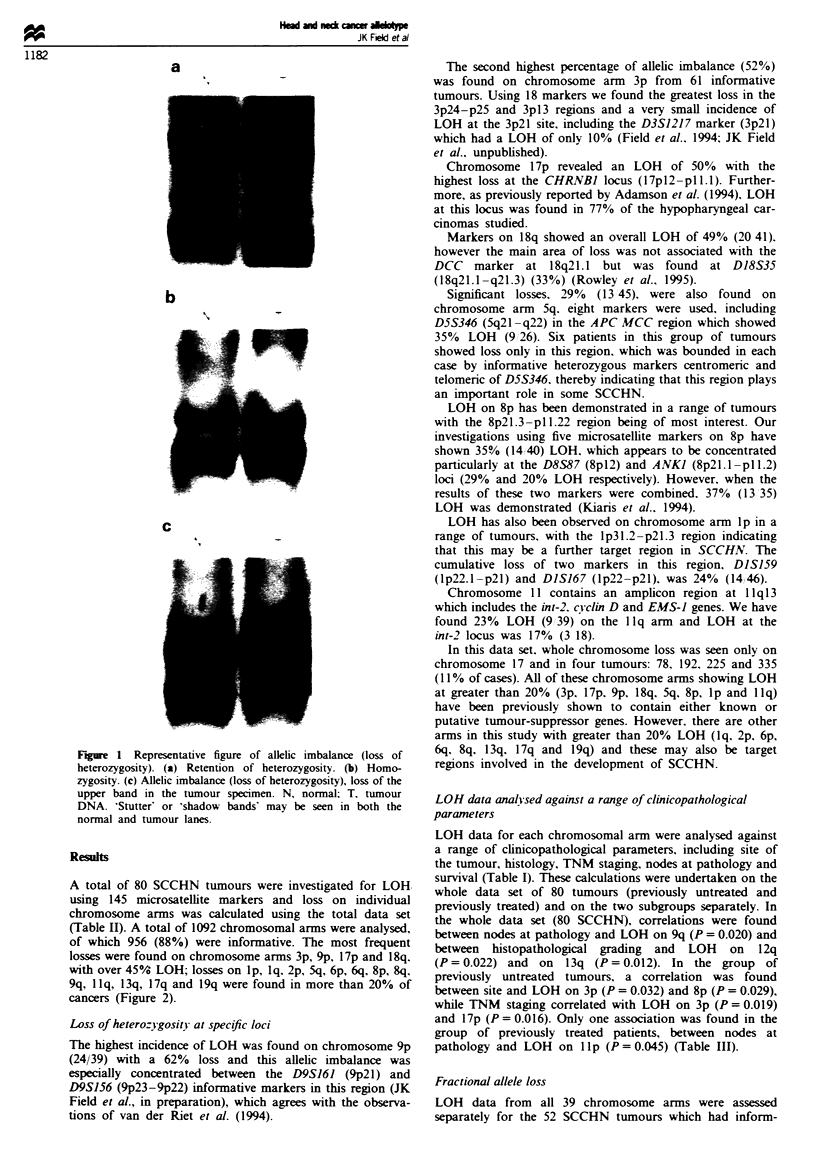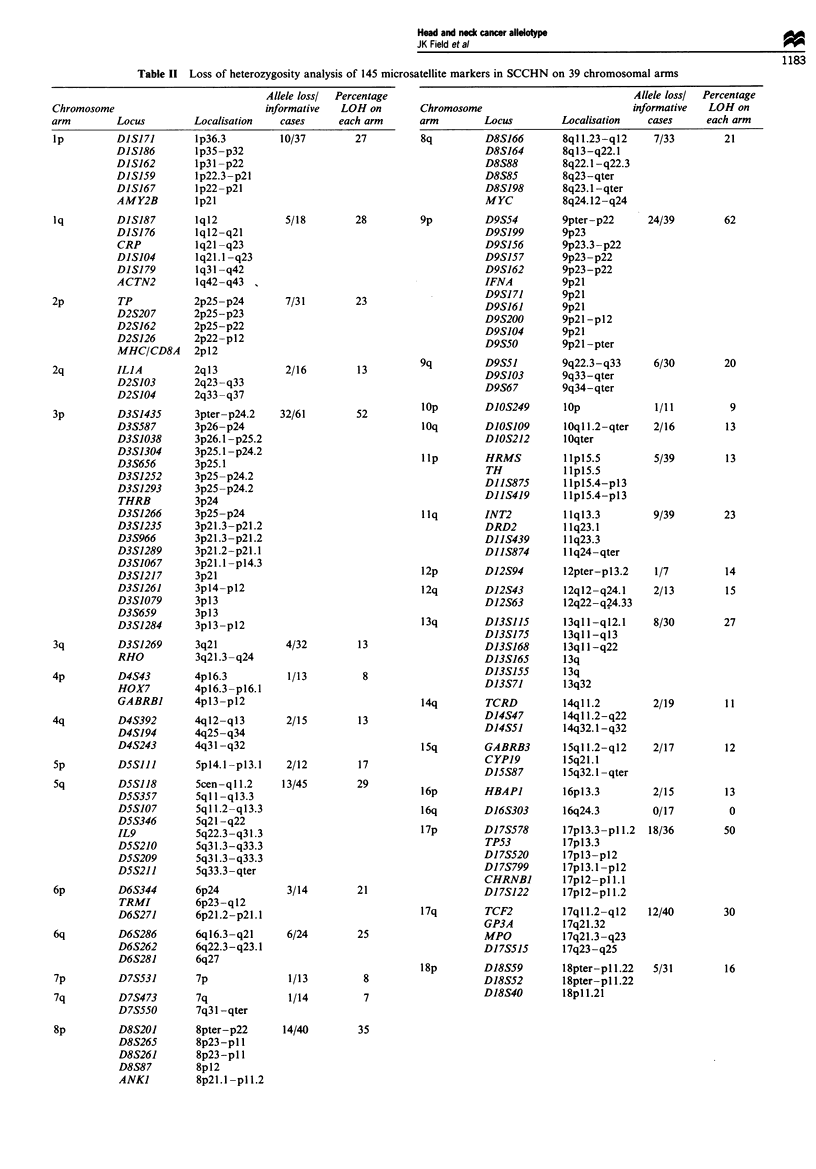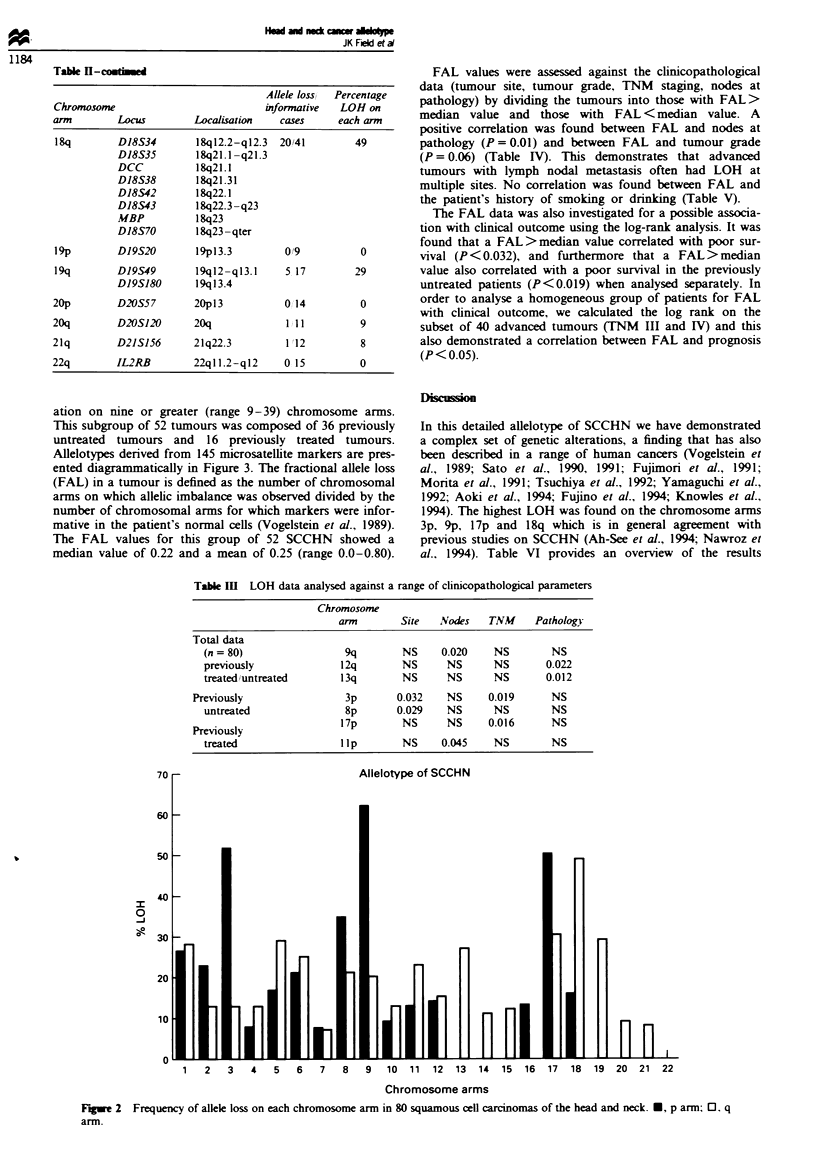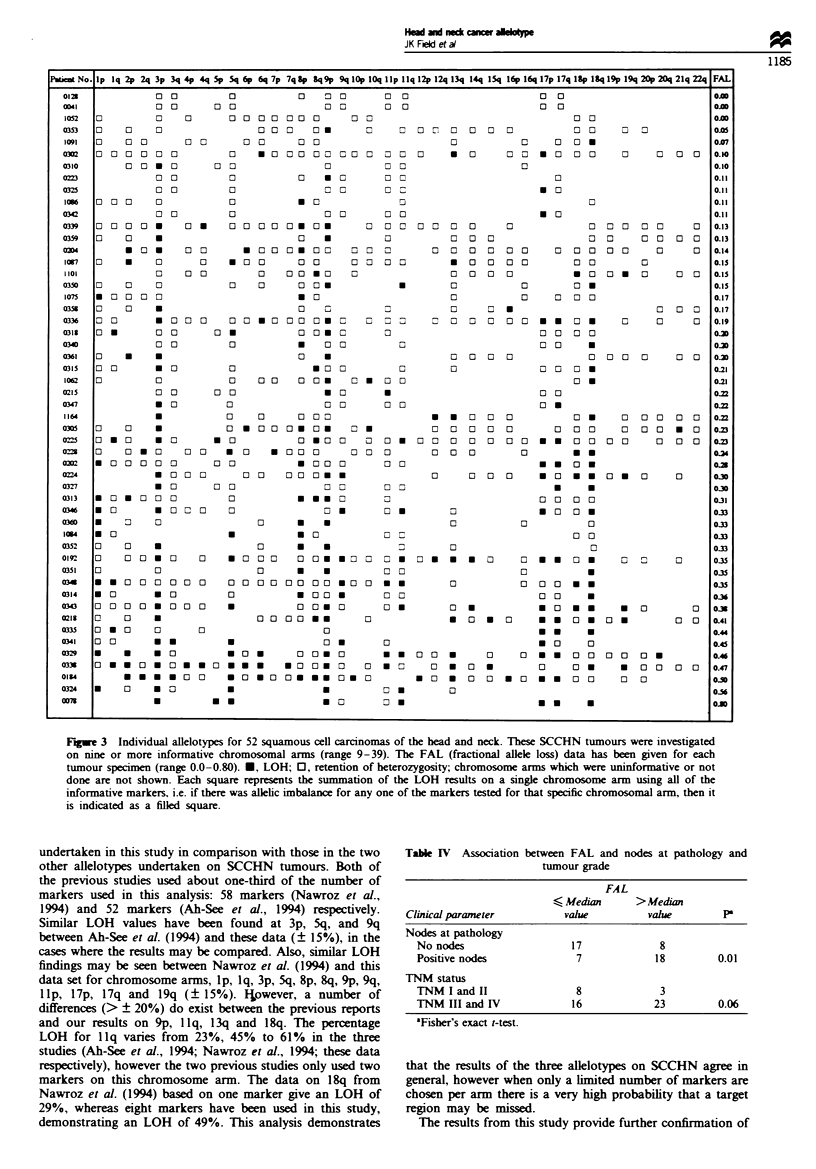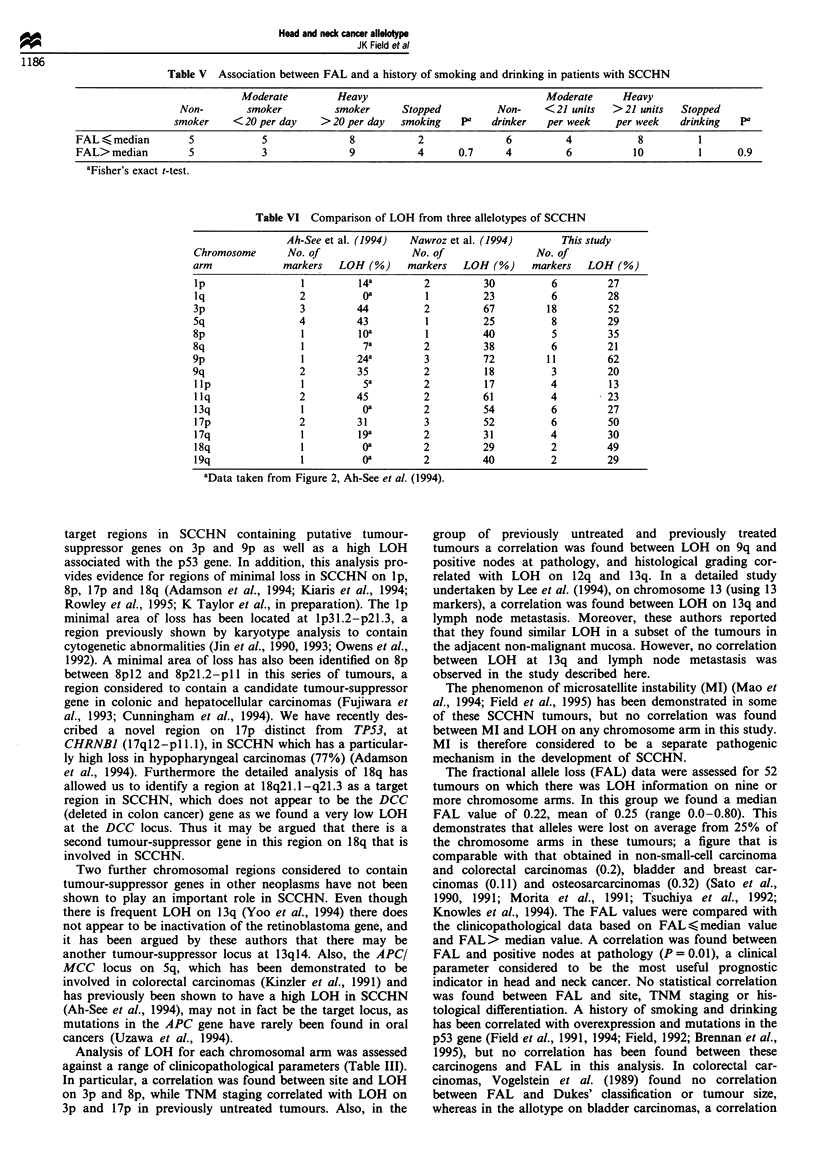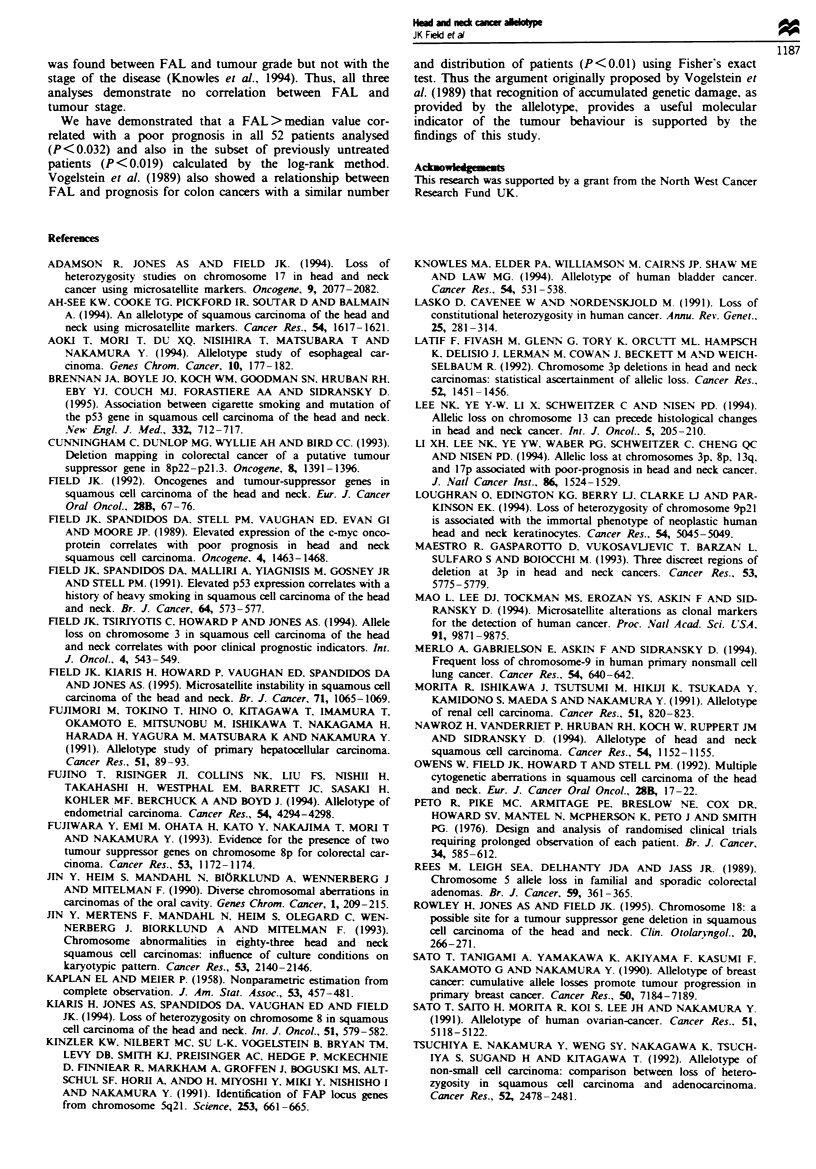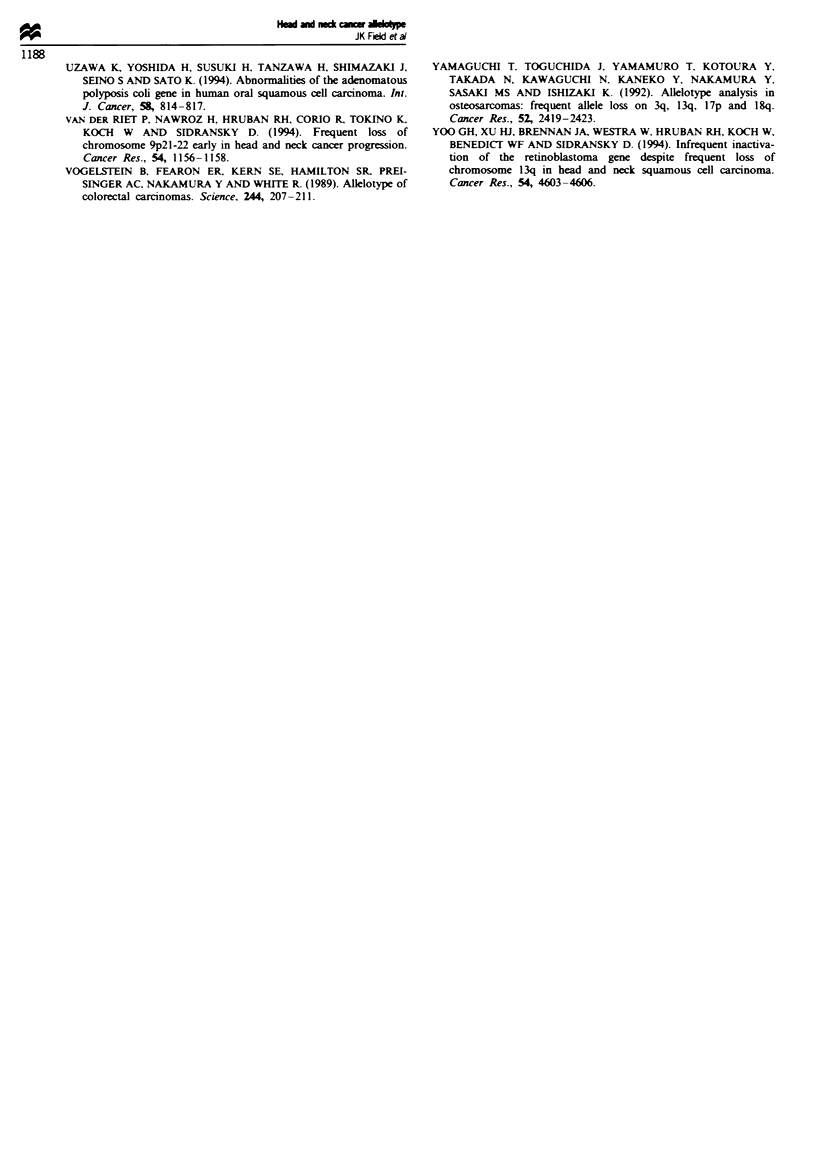# Allelotype of squamous cell carcinoma of the head and neck: fractional allele loss correlates with survival.

**DOI:** 10.1038/bjc.1995.483

**Published:** 1995-11

**Authors:** J. K. Field, H. Kiaris, J. M. Risk, C. Tsiriyotis, R. Adamson, V. Zoumpourlis, H. Rowley, K. Taylor, J. Whittaker, P. Howard

**Affiliations:** Department of Clinical Dental Sciences, University of Liverpool, UK.

## Abstract

**Images:**


British Journal of Cancer (1995) 72, 1180-1188

© 1995 Stockton Press All rights reserved 0007-0920/95 $12.00

### Allelotype of squamous cell carcinoma of the head and neck: fractional allele loss correlates with survival

JK Field[1], H Kiaris[1,2], JM Risk[1], C Tsiriyotis[1,2], R Adamson[1], V Zoumpourlis[1,2], H Rowley[1,3], K Taylor[1], J Whittaker[4], P Howard[5], JC Beirne[6], JR Gosney[7], J Woolgar[1], ED Vaughan[1,6], DA Spandidos[2] and AS Jones[3]

[1]*Molecular Genetics and Oncology Group, Department of Clinical Dental Sciences, The University of Liverpool, Liverpool L69 3BX, UK;* [2]*National Hellenic Research Foundation, 48 Vas. Constantinou Avenue, GR-11635 Athens, Greece;* [3]*Department of Otorhinolaryngology, The University of Liverpool, Liverpool L69 3BX, UK;* [4]*Regional Molecular Genetics Laboratory, Alder Hey Children's Hospital, Liverpool L12 2AP, UK;* [5]*Regional Cytogenetics Unit, Royal Liverpool University Hospital, Liverpool, UK;* [6]*Maxillofacial Unit, Walton Hospital, Rice Lane, Liverpool, UK;* [7]*Department of Pathology, The University of Liverpool, Liverpool L69 3BX, UK.*

**Summary** Allelic imbalance or loss of heterozygosity (LOH) studies have been used extensively to identify regions on chromosomes that may contain putative tumour-suppressor genes. We have undertaken an extensive allelotype of 80 specimens of squamous cell carcinoma of the head and neck (SCCHN) using 145 polymorphic microsatellite markers on 39 chromosome arms. Allelic imbalances were found most frequently on chromosome arms 3p, 9p, 17p and 18q with over 45% LOH and imbalances on 1p, 1q, 2p, 5q, 6p, 6q, 8p, 8q, 9q, 11q, 13q, 17q and 19q were found in more than 20% of SCCHN. These LOH data were analysed against a range of clinicopathological parameters which included previously untreated and previously treated tumours; correlations were found between LOH on 9q and nodes at pathology ($P = 0.02$) and between histopathological grade and LOH on 12q ($P = 0.02$) and 13q ($P = 0.01$). In the group of previously untreated tumours, a correlation was found between site of tumour and LOH on 3p ($P = 0.019$), and 8p ($P = 0.029$), while TNM staging correlated with LOH on 3p ($P = 0.019$) and 17p ($P = 0.016$). Fractional allele loss (FAL) was calculated for 52 tumours with LOH data on nine or more chromosomal arms and found to have a median value of 0.22 (range 0.0-0.80). Correlations were found between FAL > median value and nodes at pathology ($P = 0.01$) and tumour grade ($P = 0.06$), demonstrating that advanced tumours with lymph node metastasis often had LOH at multiple sites. FAL > median value was found to correlate with a poor survival ($P < 0.03$) and, furthermore, FAL > median value correlated with poor survival in the previously untreated patients ($P < 0.019$). These results indicate that assessment of the accumulation of genetic damage, as provided by allelotype data, provides a useful molecular indicator of the tumour behaviour and clinical outcome.

**Keywords**: head and neck cancer; oral cancer; allelotype; microsatellites; loss of heterozygosity; fractional allele loss

The chromosomal locations of many different putative human tumour-suppressor genes have been identified by loss of heterozygosity (LOH) studies (Rees *et al.*, 1989; Lasko *et al.*, 1991; Latif *et al.*, 1992; Cunningham *et al.*, 1993; Adamson *et al.*, 1994). A number of oncogenic and tumour-suppressor gene functions have been demonstrated in squamous cell carcinoma of the head and neck (SCCHN) (Field *et al.*, 1989, 1991; Field, 1992), and the results of LOH analysis currently point to several novel tumour-suppressor gene sites in this disease (Maestro *et al.*, 1993; Adamson *et al.*, 1994; Ah-See *et al.*, 1994; Field *et al.*, 1994; Kiaris *et al.*, 1994; Li *et al.*, 1994; Loughran *et al.*, 1994; Merlo *et al.*, 1994; Nawroz *et al.*, 1994; van der Reit *et al.*, 1994).

To date only two global analyses of the whole genome have been undertaken with the view to determine the fractional allele loss (FAL) of specific tumours and thus provide information concerning the 'genetic burden' of the disease during its progression as measured by clinicopathological parameters and survival data. This type of analysis has been undertaken in colorectal (Vogelstein *et al.*, 1989) and bladder cancers (Knowles *et al.*, 1994), and provides an indication of interacting genetic mechanisms in the development of these diseases. In addition, the results of such detailed allelotypes may aid the interpretation of carcinogenesis and the development of molecular progression models for specific tumours.

We have undertaken a very comprehensive allelotype of SCCHN using 145 microsatellite markers in order to identify common regions of allelic imbalance and to analyse the interactions of these regions by calculating the fractional allele loss (FAL) in these tumours.

Correspondence: JK Field
Received 13 January 1995; revised 25 May 1995; accepted 16 June 1995.

### Materials and methods

### *Specimens*

Eighty SCCHN tumour specimens were collected at the Royal Liverpool University Hospital, Department of Otorhinolaryngology and at the Walton Hospital Liverpool, Maxillofacial Unit. Tumour samples obtained from surgical specimens were frozen in liquid nitrogen and stored at −70°C. The clinicopathological data on the 52 SCCHN used in fractional allele loss analysis is given in Table I. This group of patients had LOH information on nine or greater (9-39) chromosome arms.

### *DNA extraction*

All the tumour specimens used for LOH analysis were microdissected to yield at least 60% tumour cells before DNA preparation. Genomic DNA was extracted from tumour specimens using the Nucleon II DNA extraction kit (Scotlab) following the manufacturer's instructions. Genomic DNA samples were stored at 4°C.

### *PCR and LOH analysis*

Microsatellite repeat primers were obtained from Isogen (The Netherlands). PCR reactions were performed in a 25 μl reaction volume and contained 200 ng of genomic DNA, 200 μM each dNTP, 5 pmol each of forward and reverse primers,

**Table I** Clinicopathological characteristics of the 52 squamous cell carinomas investigated on nine or more chromosomal arms

| *Patient number* | *History* | *TNM* | *Histology* | *Nodes at pathology* | *Survival (months)* | *Fate* | *FAL*[a] *≤ Median* |
|---|---|---|---|---|---|---|---|
| 0128 | PT | TI | Moderate | − ve | 8 | Died of disease | 0.00 |
| 0041 | PU | TII | Well | − ve | 35 | Alive | 0.00 |
| 1052 | PU | TIV | Moderate | − ve | 29 | Alive | 0.00 |
| 0353 | PU | TIV | Moderate | − ve | 12 | Died of disease | 0.05 |
| 1091 | PU | TIII | Well | − ve | 21 | Alive | 0.07 |
| 0302 | PU | TIII | Moderate | − ve | 32 | Alive | 0.10 |
| 0310 | PT | TIV | Moderate | − ve | 5 | Alive | 0.10 |
| 0223 | PT | TI | Poor | − ve | 44 | Alive | 0.11 |
| 0325 | PU | TIV | Moderate | + ve | 27 | Alive | 0.11 |
| 1086 | PU | TIV | Poor | + ve | 17 | Alive | 0.11 |
| 0342 | PU | TIII | Moderate | + ve | 18 | Alive | 0.11 |
| 0339 | PT | TI | Moderate | − ve | 68 | Alive | 0.13 |
| 0359 | PT | TIII | Moderate | − ve | 12 | Alive | 0.13 |
| 0204 | PU | TIII | Moderate | − ve | 21 | Died of disease | 0.14 |
| 1087 | PU | TIV | Moderate | − ve | 22 | Alive | 0.15 |
| 1101 | PU | TII | Well | − ve | 17 | Alive | 0.15 |
| 0350 | PT | TIII | Moderate | + ve | 11 | Alive | 0.15 |
| 1075 | PU | TII | Moderate | − ve | 23 | Alive | 0.17 |
| 0358 | PU | TIV | Moderate | + ve | 12 | Alive | 0.17 |
| 0336 | PU | TIII | Well | − ve | 2 | Died of disease | 0.19 |
| 0318 | PT | TIII | Moderate | − ve | 34 | Alive | 0.20 |
| 0340 | PT | TIII | Moderate | − ve | 10 | Alive | 0.20 |
| 0361 | PU | TII | Moderate | + ve | 8 | Alive | 0.20 |
| 0315 | PU | TIV | Moderate | + ve | 99 | Alive | 0.21 |
| 1062 | PU | TI | Moderate | No data | 7 | Alive | 0.21 |
| | | | | | | | *> Median* |
| 0215 | PU | TI | Well | − ve | 36 | Alive | 0.22 |
| 0347 | PT | TIII | Moderate | − ve | 48 | Alive | 0.22 |
| 1164 | PT | TIV | Poor | − ve | 21 | Died of disease | 0.22 |
| 0305 | PU | TIV | Moderate | + ve | 13 | Died of disease | 0.23 |
| 0225 | PT | TIII | Moderate | − ve | 87 | Died of disease | 0.23 |
| 0228 | PT | TIV | Poor | No data | 56 | Alive | 0.24 |
| 0202 | PT | | Moderate | + ve | 74 | Alive | 0.28 |
| 0224 | PU | TIII | Moderate | + ve | 20 | Alive | 0.30 |
| 0327 | PU | TIV | Moderate | + ve | 25 | Alive | 0.30 |
| 0313 | PU | TIV | Moderate | + ve | 8 | Alive | 0.31 |
| 0346 | PU | TIV | Moderate | + ve | 17 | Died of disease | 0.33 |
| 0360 | PU | TIV | Moderate | + ve | 9 | DOC | 0.33 |
| 1084 | PU | TII | Well | − ve | 9 | Died of disease | 0.33 |
| 0352 | PU | TIV | Moderate | + ve | 11 | Alive | 0.33 |
| 0192 | PU | TIV | Poor | + ve | 14 | Died of disease | 0.35 |
| 0351 | PU | TIV | Moderate | − ve | 11 | Alive | 0.35 |
| 0348 | PT | TIII | Not defined | + ve | 15 | Alive | 0.35 |
| 0314 | PU | TIV | Moderate | + ve | 8 | Died of disease | 0.36 |
| 0343 | PU | TIV | Moderate | + ve | 19 | Alive | 0.38 |
| 0218 | PU | TIV | Poor | + ve | 5 | Died of disease | 0.41 |
| 0335 | PT | TI | Moderate | + ve | 18 | Alive | 0.44 |
| 0341 | PU | TIII | Moderate | + ve | 0 | Died of disease | 0.45 |
| 0329 | PU | TIV | Moderate | + ve | 7 | Alive | 0.46 |
| 0338 | PU | TIV | Moderate | − ve | 20 | Alive | 0.47 |
| 0184 | PU | TIII | Poor | + ve | 16 | Died of disease | 0.50 |
| 0324 | PU | TIV | Moderate | + ve | 12 | Alive | 0.56 |
| 0078 | PT | TIII | Poor | − ve | 22 | Died of disease | 0.80 |

PU, previously untreated; PT, previously treated; DOC, died of other causes. [a]FAL, fractional allele loss (number of chromosome arms lost divided by the number of chromosome arms examined). Median value of FAL in this group of SCCHN was 0.22.

0.5 units of *Taq* polymerase (Advanced Biotechnologies) and 2.5 μl 10 × buffer [670 mM Tris-HCl, pH 8.5; 166 mM ammonium sulphate; 67 mM magnesium chloride; 1.7 mg $ml^{-1}$ bovine serum albumin (BSA); 100 μM β-mercaptoethanol; 1% (w/v) Triton X-100]. The reactions were denatured for 5 min at 95°C then the DNA was amplified for 30 cycles of 95°C for 30 s, 57°C for 30 s and 72°C for 30 s. Aliquots of 10 μl of the PCR product were electrophoresed for 10 h on a 10% non-denaturing polyacrylamide gel at 250 V and visualised by silver staining.

LOH or allelic imbalance was scored by direct visual comparison of the allelic ratios of the normal and tumour specimens. Examples of heterozygous, homozygous and LOH in tumour/normal SCCHN specimens are given in Figure 1. Complete loss or reduced intensity of one allele in the tumour was considered as LOH. In the cases where there was only a reduced intensity of one allele this was considered to be due to contamination of tumour tissue by normal stroma. It has been previously noted by Ah-See *et al.* (1994) that the PCR techniques used by a number of authors in similar studies cannot readily distinguish between allelic duplication or low-level amplification leading to loss of heterozygosity. This caveat has to be taken into consideration when interpreting these results and thus LOH may not necessarily be indicative of the presence of a tumour-suppressor gene.

### *Statistical analysis*

Quantitative data were analysed by $\chi^2$ or Fisher's exact test where appropriate. Survival curves were drawn using the Kaplan–Meier (1958) product limit estimate. Differences between survival times were analysed by the log-rank method (Peto *et al.*, 1976).

**Figure 1** Representative figure of allelic imbalance (loss of heterozygosity). (**a**) Retention of heterozygosity. (**b**) Homozygosity. (**c**) Allelic imbalance (loss of heterozygosity), loss of the upper band in the tumour specimen. N, normal; T, tumour DNA. 'Stutter' or 'shadow bands' may be seen in both the normal and tumour lanes.

### Results

A total of 80 SCCHN tumours were investigated for LOH using 145 microsatellite markers and loss on individual chromosome arms was calculated using the total data set (Table II). A total of 1092 chromosomal arms were analysed, of which 956 (88%) were informative. The most frequent losses were found on chromosome arms 3p, 9p, 17p and 18q, with over 45% LOH; losses on 1p, 1q, 2p, 5q, 6p, 6q, 8p, 8q, 9q, 11q, 13q, 17q and 19q were found in more than 20% of cancers (Figure 2).

### *Loss of heterozygosity at specific loci*

The highest incidence of LOH was found on chromosome 9p (24/39) with a 62% loss and this allelic imbalance was especially concentrated between the *D9S161* (9p21) and *D9S156* (9p23–9p22) informative markers in this region (JK Field *et al*., in preparation), which agrees with the observations of van der Riet *et al*. (1994).

The second highest percentage of allelic imbalance (52%) was found on chromosome arm 3p from 61 informative tumours. Using 18 markers we found the greatest loss in the 3p24–p25 and 3p13 regions and a very small incidence of LOH at the 3p21 site, including the *D3S1217* marker (3p21) which had a LOH of only 10% (Field *et al*., 1994; JK Field *et al*., unpublished).

Chromosome 17p revealed an LOH of 50% with the highest loss at the *CHRNB1* locus (17p12–p11.1). Furthermore, as previously reported by Adamson *et al*. (1994), LOH at this locus was found in 77% of the hypopharyngeal carcinomas studied.

Markers on 18q showed an overall LOH of 49% (20/41), however the main area of loss was not associated with the *DCC* marker at 18q21.1 but was found at *D18S35* (18q21.1–q21.3) (33%) (Rowley *et al*., 1995).

Significant losses, 29% (13/45), were also found on chromosome arm 5q, eight markers were used, including *D5S346* (5q21–q22) in the *APC/MCC* region which showed 35% LOH (9/26). Six patients in this group of tumours showed loss only in this region, which was bounded in each case by informative heterozygous markers centromeric and telomeric of *D5S346*, thereby indicating that this region plays an important role in some SCCHN.

LOH on 8p has been demonstrated in a range of tumours with the 8p21.3–p11.22 region being of most interest. Our investigations using five microsatellite markers on 8p have shown 35% (14/40) LOH, which appears to be concentrated particularly at the *D8S87* (8p12) and *ANK1* (8p21.1–p11.2) loci (29% and 20% LOH respectively). However, when the results of these two markers were combined, 37% (13/35) LOH was demonstrated (Kiaris *et al*., 1994).

LOH has also been observed on chromosome arm 1p in a range of tumours, with the 1p31.2–p21.3 region indicating that this may be a further target region in *SCCHN*. The cumulative loss of two markers in this region, *D1S159* (1p22.1–p21) and *D1S167* (1p22–p21), was 24% (14/46).

Chromosome 11 contains an amplicon region at 11q13 which includes the *int-2*, *cyclin D* and *EMS-1* genes. We have found 23% LOH (9/39) on the 11q arm and LOH at the *int-2* locus was 17% (3/18).

In this data set, whole chromosome loss was seen only on chromosome 17 and in four tumours: 78, 192, 225 and 335 (11% of cases). All of these chromosome arms showing LOH at greater than 20% (3p, 17p, 9p, 18q, 5q, 8p, 1p and 11q) have been previously shown to contain either known or putative tumour-suppressor genes. However, there are other arms in this study with greater than 20% LOH (1q, 2p, 6p, 6q, 8q, 13q, 17q and 19q) and these may also be target regions involved in the development of SCCHN.

### *LOH data analysed against a range of clinicopathological parameters*

LOH data for each chromosomal arm were analysed against a range of clinicopathological parameters, including site of the tumour, histology, TNM staging, nodes at pathology and survival (Table I). These calculations were undertaken on the whole data set of 80 tumours (previously untreated and previously treated) and on the two subgroups separately. In the whole data set (80 SCCHN), correlations were found between nodes at pathology and LOH on 9q ($P = 0.020$) and between histopathological grading and LOH on 12q ($P = 0.022$) and on 13q ($P = 0.012$). In the group of previously untreated tumours, a correlation was found between site and LOH on 3p ($P = 0.032$) and 8p ($P = 0.029$), while TNM staging correlated with LOH on 3p ($P = 0.019$) and 17p ($P = 0.016$). Only one association was found in the group of previously treated patients, between nodes at pathology and LOH on 11p ($P = 0.045$) (Table III).

### *Fractional allele loss*

LOH data from all 39 chromosome arms were assessed separately for the 52 SCCHN tumours which had inform-

**Table II** Loss of heterozygosity analysis of 145 microsatellite markers in SCCHN on 39 chromosomal arms

| Chromosome arm | Locus | Localisation | Allele loss/ informative cases | Percentage LOH on each arm |
|---|---|---|---|---|
| 1p | *D1S171* | 1p36.3 | 10/37 | 27 |
| | *D1S186* | 1p35-p32 | | |
| | *D1S162* | 1p31-p22 | | |
| | *D1S159* | 1p22.3-p21 | | |
| | *D1S167* | 1p22-p21 | | |
| | *AMY2B* | 1p21 | | |
| 1q | *D1S187* | 1q12 | 5/18 | 28 |
| | *D1S176* | 1q12-q21 | | |
| | *CRP* | 1q21-q23 | | |
| | *D1S104* | 1q21.1-q23 | | |
| | *D1S179* | 1q31-q42 | | |
| | *ACTN2* | 1q42-q43 | | |
| 2p | *TP* | 2p25-p24 | 7/31 | 23 |
| | *D2S207* | 2p25-p23 | | |
| | *D2S162* | 2p25-p22 | | |
| | *D2S126* | 2p22-p12 | | |
| | *MHC/CD8A* | 2p12 | | |
| 2q | *IL1A* | 2q13 | 2/16 | 13 |
| | *D2S103* | 2q23-q33 | | |
| | *D2S104* | 2q33-q37 | | |
| 3p | *D3S1435* | 3pter-p24.2 | 32/61 | 52 |
| | *D3S587* | 3p26-p24 | | |
| | *D3S1038* | 3p26.1-p25.2 | | |
| | *D3S1304* | 3p25.1-p24.2 | | |
| | *D3S656* | 3p25.1 | | |
| | *D3S1252* | 3p25-p24.2 | | |
| | *D3S1293* | 3p25-p24.2 | | |
| | *THRB* | 3p24 | | |
| | *D3S1266* | 3p25-p24 | | |
| | *D3S1235* | 3p21.3-p21.2 | | |
| | *D3S966* | 3p21.3-p21.2 | | |
| | *D3S1289* | 3p21.2-p21.1 | | |
| | *D3S1067* | 3p21.1-p14.3 | | |
| | *D3S1217* | 3p21 | | |
| | *D3S1261* | 3p14-p12 | | |
| | *D3S1079* | 3p13 | | |
| | *D3S659* | 3p13 | | |
| | *D3S1284* | 3p13-p12 | | |
| 3q | *D3S1269* | 3q21 | 4/32 | 13 |
| | *RHO* | 3q21.3-q24 | | |
| 4p | *D4S43* | 4p16.3 | 1/13 | 8 |
| | *HOX7* | 4p16.3-p16.1 | | |
| | *GABRB1* | 4p13-p12 | | |
| 4q | *D4S392* | 4q12-q13 | 2/15 | 13 |
| | *D4S194* | 4q25-q34 | | |
| | *D4S243* | 4q31-q32 | | |
| 5p | *D5S111* | 5p14.1-p13.1 | 2/12 | 17 |
| 5q | *D5S118* | 5cen-q11.2 | 13/45 | 29 |
| | *D5S357* | 5q11-q13.3 | | |
| | *D5S107* | 5q11.2-q13.3 | | |
| | *D5S346* | 5q21-q22 | | |
| | *IL9* | 5q22.3-q31.3 | | |
| | *D5S210* | 5q31.3-q33.3 | | |
| | *D5S209* | 5q31.3-q33.3 | | |
| | *D5S211* | 5q33.3-qter | | |
| 6p | *D6S344* | 6p24 | 3/14 | 21 |
| | *TRM1* | 6p23-q12 | | |
| | *D6S271* | 6p21.2-p21.1 | | |
| 6q | *D6S286* | 6q16.3-q21 | 6/24 | 25 |
| | *D6S262* | 6q22.3-q23.1 | | |
| | *D6S281* | 6q27 | | |
| 7p | *D7S531* | 7p | 1/13 | 8 |
| 7q | *D7S473* | 7q | 1/14 | 7 |
| | *D7S550* | 7q31-qter | | |
| 8p | *D8S201* | 8pter-p22 | 14/40 | 35 |
| | *D8S265* | 8p23-p11 | | |
| | *D8S261* | 8p23-p11 | | |
| | *D8S87* | 8p12 | | |
| | *ANK1* | 8p21.1-p11.2 | | |
| 8q | *D8S166* | 8q11.23-q12 | 7/33 | 21 |
| | *D8S164* | 8q13-q22.1 | | |
| | *D8S88* | 8q22.1-q22.3 | | |
| | *D8S85* | 8q23-qter | | |
| | *D8S198* | 8q23.1-qter | | |
| | *MYC* | 8q24.12-q24 | | |
| 9p | *D9S54* | 9pter-p22 | 24/39 | 62 |
| | *D9S199* | 9p23 | | |
| | *D9S156* | 9p23.3-p22 | | |
| | *D9S157* | 9p23-p22 | | |
| | *D9S162* | 9p23-p22 | | |
| | *IFNA* | 9p21 | | |
| | *D9S171* | 9p21 | | |
| | *D9S161* | 9p21 | | |
| | *D9S200* | 9p21-p12 | | |
| | *D9S104* | 9p21 | | |
| | *D9S50* | 9p21-pter | | |
| 9q | *D9S51* | 9q22.3-q33 | 6/30 | 20 |
| | *D9S103* | 9q33-qter | | |
| | *D9S67* | 9q34-qter | | |
| 10p | *D10S249* | 10p | 1/11 | 9 |
| 10q | *D10S109* | 10q11.2-qter | 2/16 | 13 |
| | *D10S212* | 10qter | | |
| 11p | *HRMS* | 11p15.5 | 5/39 | 13 |
| | *TH* | 11p15.5 | | |
| | *D11S875* | 11p15.4-p13 | | |
| | *D11S419* | 11p15.4-p13 | | |
| 11q | *INT2* | 11q13.3 | 9/39 | 23 |
| | *DRD2* | 11q23.1 | | |
| | *D11S439* | 11q23.3 | | |
| | *D11S874* | 11q24-qter | | |
| 12p | *D12S94* | 12pter-p13.2 | 1/7 | 14 |
| 12q | *D12S43* | 12q12-q24.1 | 2/13 | 15 |
| | *D12S63* | 12q22-q24.33 | | |
| 13q | *D13S115* | 13q11-q12.1 | 8/30 | 27 |
| | *D13S175* | 13q11-q13 | | |
| | *D13S168* | 13q11-q22 | | |
| | *D13S165* | 13q | | |
| | *D13S155* | 13q | | |
| | *D13S71* | 13q32 | | |
| 14q | *TCRD* | 14q11.2 | 2/19 | 11 |
| | *D14S47* | 14q11.2-q22 | | |
| | *D14S51* | 14q32.1-q32 | | |
| 15q | *GABRB3* | 15q11.2-q12 | 2/17 | 12 |
| | *CYP19* | 15q21.1 | | |
| | *D15S87* | 15q32.1-qter | | |
| 16p | *HBAP1* | 16p13.3 | 2/15 | 13 |
| 16q | *D16S303* | 16q24.3 | 0/17 | 0 |
| 17p | *D17S578* | 17p13.3-p11.2 | 18/36 | 50 |
| | *TP53* | 17p13.3 | | |
| | *D17S520* | 17p13-p12 | | |
| | *D17S799* | 17p13.1-p12 | | |
| | *CHRNB1* | 17p12-p11.1 | | |
| | *D17S122* | 17p12-p11.2 | | |
| 17q | *TCF2* | 17q11.2-q12 | 12/40 | 30 |
| | *GP3A* | 17q21.32 | | |
| | *MPO* | 17q21.3-q23 | | |
| | *D17S515* | 17q23-q25 | | |
| 18p | *D18S59* | 18pter-p11.22 | 5/31 | 16 |
| | *D18S52* | 18pter-p11.22 | | |
| | *D18S40* | 18p11.21 | | |

**Table II**–continued

| Chromosome arm | Locus | Localisation | Allele loss/informative cases | Percentage LOH on each arm |
|---|---|---|---|---|
| 18q | *D18S34* | 18q12.2–q12.3 | 20/41 | 49 |
| | *D18S35* | 18q21.1–q21.3 | | |
| | *DCC* | 18q21.1 | | |
| | *D18S38* | 18q21.31 | | |
| | *D18S42* | 18q22.1 | | |
| | *D18S43* | 18q22.3–q23 | | |
| | *MBP* | 18q23 | | |
| | *D18S70* | 18q23–qter | | |
| 19p | *D19S20* | 19p13.3 | 0/9 | 0 |
| 19q | *D19S49* | 19q12–q13.1 | 5/17 | 29 |
| | *D19S180* | 19q13.4 | | |
| 20p | *D20S57* | 20p13 | 0/14 | 0 |
| 20q | *D20S120* | 20q | 1/11 | 9 |
| 21q | *D21S156* | 21q22.3 | 1/12 | 8 |
| 22q | *IL2RB* | 22q11.2–q12 | 0/15 | 0 |

ation on nine or greater (range 9–39) chromosome arms. This subgroup of 52 tumours was composed of 36 previously untreated tumours and 16 previously treated tumours. Allelotypes derived from 145 microsatellite markers are presented diagrammatically in Figure 3. The fractional allele loss (FAL) in a tumour is defined as the number of chromosomal arms on which allelic imbalance was observed divided by the number of chromosomal arms for which markers were informative in the patient's normal cells (Vogelstein *et al.*, 1989). The FAL values for this group of 52 SCCHN showed a median value of 0.22 and a mean of 0.25 (range 0.0–0.80).

FAL values were assessed against the clinicopathological data (tumour site, tumour grade, TNM staging, nodes at pathology) by dividing the tumours into those with FAL > median value and those with FAL < median value. A positive correlation was found between FAL and nodes at pathology ($P = 0.01$) and between FAL and tumour grade ($P = 0.06$) (Table IV). This demonstrates that advanced tumours with lymph nodal metastasis often had LOH at multiple sites. No correlation was found between FAL and the patient's history of smoking or drinking (Table V).

The FAL data was also investigated for a possible association with clinical outcome using the log-rank analysis. It was found that a FAL > median value correlated with poor survival ($P < 0.032$), and furthermore that a FAL > median value also correlated with a poor survival in the previously untreated patients ($P < 0.019$) when analysed separately. In order to analyse a homogeneous group of patients for FAL with clinical outcome, we calculated the log rank on the subset of 40 advanced tumours (TNM III and IV) and this also demonstrated a correlation between FAL and prognosis ($P < 0.05$).

### Discussion

In this detailed allelotype of SCCHN we have demonstrated a complex set of genetic alterations, a finding that has also been described in a range of human cancers (Vogelstein *et al.*, 1989; Sato *et al.*, 1990, 1991; Fujimori *et al.*, 1991; Morita *et al.*, 1991; Tsuchiya *et al.*, 1992; Yamaguchi *et al.*, 1992; Aoki *et al.*, 1994; Fujino *et al.*, 1994; Knowles *et al.*, 1994). The highest LOH was found on the chromosome arms 3p, 9p, 17p and 18q which is in general agreement with previous studies on SCCHN (Ah-See *et al.*, 1994; Nawroz *et al.*, 1994). Table VI provides an overview of the results

**Table III** LOH data analysed against a range of clinicopathological parameters

| | Chromosome arm | Site | Nodes | TNM | Pathology |
|---|---|---|---|---|---|
| Total data | | | | | |
| ($n = 80$) | 9q | NS | 0.020 | NS | NS |
| previously | 12q | NS | NS | NS | 0.022 |
| treated/untreated | 13q | NS | NS | NS | 0.012 |
| Previously | 3p | 0.032 | NS | 0.019 | NS |
| untreated | 8p | 0.029 | NS | NS | NS |
| | 17p | NS | NS | 0.016 | NS |
| Previously treated | 11p | NS | 0.045 | NS | NS |

**Figure 2** Frequency of allele loss on each chromosome arm in 80 squamous cell carcinomas of the head and neck. ■, p arm; □, q arm.

**Figure 3** Individual allelotypes for 52 squamous cell carcinomas of the head and neck. These SCCHN tumours were investigated on nine or more informative chromosomal arms (range 9–39). The FAL (fractional allele loss) data has been given for each tumour specimen (range 0.0–0.80). ■, LOH; □, retention of heterozygosity; chromosome arms which were uninformative or not done are not shown. Each square represents the summation of the LOH results on a single chromosome arm using all of the informative markers, i.e. if there was allelic imbalance for any one of the markers tested for that specific chromosomal arm, then it is indicated as a filled square.

undertaken in this study in comparison with those in the two other allelotypes undertaken on SCCHN tumours. Both of the previous studies used about one-third of the number of markers used in this analysis: 58 markers (Nawroz *et al.*, 1994) and 52 markers (Ah-See *et al.*, 1994) respectively. Similar LOH values have been found at 3p, 5q, and 9q between Ah-See *et al.* (1994) and these data (± 15%), in the cases where the results may be compared. Also, similar LOH findings may be seen between Nawroz *et al.* (1994) and this data set for chromosome arms, 1p, 1q, 3p, 5q, 8p, 8q, 9p, 9q, 11p, 17p, 17q and 19q (± 15%). However, a number of differences (> ± 20%) do exist between the previous reports and our results on 9p, 11q, 13q and 18q. The percentage LOH for 11q varies from 23%, 45% to 61% in the three studies (Ah-See *et al.*, 1994; Nawroz *et al.*, 1994; these data respectively), however the two previous studies only used two markers on this chromosome arm. The data on 18q from Nawroz *et al.* (1994) based on one marker give an LOH of 29%, whereas eight markers have been used in this study, demonstrating an LOH of 49%. This analysis demonstrates that the results of the three allelotypes on SCCHN agree in general, however when only a limited number of markers are chosen per arm there is a very high probability that a target region may be missed.

**Table IV** Association between FAL and nodes at pathology and tumour grade

| *Clinical parameter* | *FAL* ≤ *Median value* | *FAL* > *Median value* | P[a] |
|---|---|---|---|
| Nodes at pathology | | | |
| No nodes | 17 | 8 | |
| Positive nodes | 7 | 18 | 0.01 |
| TNM status | | | |
| TNM I and II | 8 | 3 | |
| TNM III and IV | 16 | 23 | 0.06 |

[a]Fisher's exact *t*-test.

The results from this study provide further confirmation of

**Table V** Association between FAL and a history of smoking and drinking in patients with SCCHN

| | *Non-smoker* | *Moderate smoker <20 per day* | *Heavy smoker >20 per day* | *Stopped smoking* | P[a] | *Non-drinker* | *Moderate <21 units per week* | *Heavy >21 units per week* | *Stopped drinking* | P[a] |
|---|---|---|---|---|---|---|---|---|---|---|
| FAL ⩽ median | 5 | 5 | 8 | 2 | | 6 | 4 | 8 | 1 | |
| FAL > median | 5 | 3 | 9 | 4 | 0.7 | 4 | 6 | 10 | 1 | 0.9 |

[a]Fisher's exact *t*-test.

**Table VI** Comparison of LOH from three allelotypes of SCCHN

| *Chromosome arm* | *Ah-See* et al. *(1994)* No. of markers | LOH (%) | *Nawroz* et al. *(1994)* No. of markers | LOH (%) | *This study* No. of markers | LOH (%) |
|---|---|---|---|---|---|---|
| 1p | 1 | 14[a] | 2 | 30 | 6 | 27 |
| 1q | 2 | 0[a] | 1 | 23 | 6 | 28 |
| 3p | 3 | 44 | 2 | 67 | 18 | 52 |
| 5q | 4 | 43 | 1 | 25 | 8 | 29 |
| 8p | 1 | 10[a] | 1 | 40 | 5 | 35 |
| 8q | 1 | 7[a] | 2 | 38 | 6 | 21 |
| 9p | 1 | 24[a] | 3 | 72 | 11 | 62 |
| 9q | 2 | 35 | 2 | 18 | 3 | 20 |
| 11p | 1 | 5[a] | 2 | 17 | 4 | 13 |
| 11q | 2 | 45 | 2 | 61 | 4 | 23 |
| 13q | 1 | 0[a] | 2 | 54 | 6 | 27 |
| 17p | 2 | 31 | 3 | 52 | 6 | 50 |
| 17q | 1 | 19[a] | 2 | 31 | 4 | 30 |
| 18q | 1 | 0[a] | 2 | 29 | 2 | 49 |
| 19q | 1 | 0[a] | 2 | 40 | 2 | 29 |

[a]Data taken from Figure 2, Ah-See *et al.* (1994).

target regions in SCCHN containing putative tumour-suppressor genes on 3p and 9p as well as a high LOH associated with the p53 gene. In addition, this analysis provides evidence for regions of minimal loss in SCCHN on 1p, 8p, 17p and 18q (Adamson *et al.*, 1994; Kiaris *et al.*, 1994; Rowley *et al.*, 1995; K Taylor *et al.*, in preparation). The 1p minimal area of loss has been located at 1p31.2–p21.3, a region previously shown by karyotype analysis to contain cytogenetic abnormalities (Jin *et al.*, 1990, 1993; Owens *et al.*, 1992). A minimal area of loss has also been identified on 8p between 8p12 and 8p21.2–p11 in this series of tumours, a region considered to contain a candidate tumour-suppressor gene in colonic and hepatocellular carcinomas (Fujiwara *et al.*, 1993; Cunningham *et al.*, 1994). We have recently described a novel region on 17p distinct from *TP53*, at *CHRNB1* (17q12–p11.1), in SCCHN which has a particularly high loss in hypopharyngeal carcinomas (77%) (Adamson *et al.*, 1994). Furthermore the detailed analysis of 18q has allowed us to identify a region at 18q21.1–q21.3 as a target region in SCCHN, which does not appear to be the *DCC* (deleted in colon cancer) gene as we found a very low LOH at the *DCC* locus. Thus it may be argued that there is a second tumour-suppressor gene in this region on 18q that is involved in SCCHN.

Two further chromosomal regions considered to contain tumour-suppressor genes in other neoplasms have not been shown to play an important role in SCCHN. Even though there is frequent LOH on 13q (Yoo *et al.*, 1994) there does not appear to be inactivation of the retinoblastoma gene, and it has been argued by these authors that there may be another tumour-suppressor locus at 13q14. Also, the *APC/MCC* locus on 5q, which has been demonstrated to be involved in colorectal carcinomas (Kinzler *et al.*, 1991) and has previously been shown to have a high LOH in SCCHN (Ah-See *et al.*, 1994), may not in fact be the target locus, as mutations in the *APC* gene have rarely been found in oral cancers (Uzawa *et al.*, 1994).

Analysis of LOH for each chromosomal arm was assessed against a range of clinicopathological parameters (Table III). In particular, a correlation was found between site and LOH on 3p and 8p, while TNM staging correlated with LOH on 3p and 17p in previously untreated tumours. Also, in the group of previously untreated and previously treated tumours a correlation was found between LOH on 9q and positive nodes at pathology, and histological grading correlated with LOH on 12q and 13q. In a detailed study undertaken by Lee *et al.* (1994), on chromosome 13 (using 13 markers), a correlation was found between LOH on 13q and lymph node metastasis. Moreover, these authors reported that they found similar LOH in a subset of the tumours in the adjacent non-malignant mucosa. However, no correlation between LOH at 13q and lymph node metastasis was observed in the study described here.

The phenomenon of microsatellite instability (MI) (Mao *et al.*, 1994; Field *et al.*, 1995) has been demonstrated in some of these SCCHN tumours, but no correlation was found between MI and LOH on any chromosome arm in this study. MI is therefore considered to be a separate pathogenic mechanism in the development of SCCHN.

The fractional allele loss (FAL) data were assessed for 52 tumours on which there was LOH information on nine or more chromosome arms. In this group we found a median FAL value of 0.22, mean of 0.25 (range 0.0–0.80). This demonstrates that alleles were lost on average from 25% of the chromosome arms in these tumours; a figure that is comparable with that obtained in non-small-cell carcinoma and colorectal carcinomas (0.2), bladder and breast carcinomas (0.11) and osteosarcarcinomas (0.32) (Sato *et al.*, 1990, 1991; Morita *et al.*, 1991; Tsuchiya *et al.*, 1992; Knowles *et al.*, 1994). The FAL values were compared with the clinicopathological data based on FAL ⩽ median value and FAL > median value. A correlation was found between FAL and positive nodes at pathology ($P = 0.01$), a clinical parameter considered to be the most useful prognostic indicator in head and neck cancer. No statistical correlation was found between FAL and site, TNM staging or histological differentiation. A history of smoking and drinking has been correlated with overexpression and mutations in the p53 gene (Field *et al.*, 1991, 1994; Field, 1992; Brennan *et al.*, 1995), but no correlation has been found between these carcinogens and FAL in this analysis. In colorectal carcinomas, Vogelstein *et al.* (1989) found no correlation between FAL and Dukes' classification or tumour size, whereas in the allotype on bladder carcinomas, a correlation was found between FAL and tumour grade but not with the stage of the disease (Knowles *et al*., 1994). Thus, all three analyses demonstrate no correlation between FAL and tumour stage.

We have demonstrated that a FAL > median value correlated with a poor prognosis in all 52 patients analysed ($P<0.032$) and also in the subset of previously untreated patients ($P<0.019$) calculated by the log-rank method. Vogelstein *et al*. (1989) also showed a relationship between FAL and prognosis for colon cancers with a similar number and distribution of patients ($P<0.01$) using Fisher's exact test. Thus the argument originally proposed by Vogelstein *et al*. (1989) that recognition of accumulated genetic damage, as provided by the allelotype, provides a useful molecular indicator of the tumour behaviour is supported by the findings of this study.

**Acknowledgements**
This research was supported by a grant from the North West Cancer Research Fund UK.

**References**

ADAMSON R. JONES AS AND FIELD JK. (1994). Loss of heterozygosity studies on chromosome 17 in head and neck cancer using microsatellite markers. *Oncogene*, **9**, 2077-2082.

AH-SEE KW. COOKE TG. PICKFORD IR. SOUTAR D AND BALMAIN A. (1994). An allelotype of squamous carcinoma of the head and neck using microsatellite markers. *Cancer Res*., **54**, 1617-1621.

AOKI T. MORI T. DU XQ. NISIHIRA T. MATSUBARA T AND NAKAMURA Y. (1994). Allelotype study of esophageal carcinoma. *Genes Chrom. Cancer*, **10**, 177-182.

BRENNAN JA. BOYLE JO. KOCH WM. GOODMAN SN. HRUBAN RH. EBY YJ. COUCH MJ. FORASTIERE AA AND SIDRANSKY D. (1995). Association between cigarette smoking and mutation of the p53 gene in squamous cell carcinoma of the head and neck. *New Engl. J. Med*., **332**, 712-717.

CUNNINGHAM C. DUNLOP MG. WYLLIE AH AND BIRD CC. (1993). Deletion mapping in colorectal cancer of a putative tumour suppressor gene in 8p22-p21.3. *Oncogene*, **8**, 1391-1396.

FIELD JK. (1992). Oncogenes and tumour-suppressor genes in squamous cell carcinoma of the head and neck. *Eur. J. Cancer Oral Oncol*., **28B**, 67-76.

FIELD JK. SPANDIDOS DA. STELL PM. VAUGHAN ED. EVAN GI AND MOORE JP. (1989). Elevated expression of the c-myc oncoprotein correlates with poor prognosis in head and neck squamous cell carcinoma. *Oncogene*, **4**, 1463-1468.

FIELD JK. SPANDIDOS DA. MALLIRI A. YIAGNISIS M. GOSNEY JR AND STELL PM. (1991). Elevated p53 expression correlates with a history of heavy smoking in squamous cell carcinoma of the head and neck. *Br. J. Cancer*, **64**, 573-577.

FIELD JK. TSIRIYOTIS C. HOWARD P AND JONES AS. (1994). Allele loss on chromosome 3 in squamous cell carcinoma of the head and neck correlates with poor clinical prognostic indicators. *Int. J. Oncol*., **4**, 543-549.

FIELD JK. KIARIS H. HOWARD P. VAUGHAN ED. SPANDIDOS DA AND JONES AS. (1995). Microsatellite instability in squamous cell carcinoma of the head and neck. *Br. J. Cancer*, **71**, 1065-1069.

FUJIMORI M. TOKINO T. HINO O. KITAGAWA T. IMAMURA T. OKAMOTO E. MITSUNOBU M. ISHIKAWA T. NAKAGAMA H. HARADA H. YAGURA M. MATSUBARA K AND NAKAMURA Y. (1991). Allelotype study of primary hepatocellular carcinoma. *Cancer Res*., **51**, 89-93.

FUJINO T. RISINGER JI. COLLINS NK. LIU FS. NISHII H. TAKAHASHI H. WESTPHAL EM. BARRETT JC. SASAKI H. KOHLER MF. BERCHUCK A AND BOYD J. (1994). Allelotype of endometrial carcinoma. *Cancer Res*., **54**, 4294-4298.

FUJIWARA Y. EMI M. OHATA H. KATO Y. NAKAJIMA T. MORI T AND NAKAMURA Y. (1993). Evidence for the presence of two tumour suppressor genes on chromosome 8p for colorectal carcinoma. *Cancer Res*., **53**, 1172-1174.

JIN Y. HEIM S. MANDAHL N. BIÖRKLUND A. WENNERBERG J AND MITELMAN F. (1990). Diverse chromosomal aberrations in carcinomas of the oral cavity. *Genes Chrom. Cancer*, **1**, 209-215.

JIN Y. MERTENS F. MANDAHL N. HEIM S. OLEGARD C. WENNERBERG J. BIORKLUND A AND MITELMAN F. (1993). Chromosome abnormalities in eighty-three head and neck squamous cell carcinomas: influence of culture conditions on karyotypic pattern. *Cancer Res*., **53**, 2140-2146.

KAPLAN EL AND MEIER P. (1958). Nonparametric estimation from complete observation. *J. Am. Stat. Assoc*., **53**, 457-481.

KIARIS H. JONES AS. SPANDIDOS DA. VAUGHAN ED AND FIELD JK. (1994). Loss of heterozygosity on chromosome 8 in squamous cell carcinoma of the head and neck. *Int. J. Oncol*., **51**, 579-582.

KINZLER KW. NILBERT MC. SU L-K. VOGELSTEIN B. BRYAN TM. LEVY DB. SMITH KJ. PREISINGER AC. HEDGE P. McKECHNIE D. FINNIEAR R. MARKHAM A. GROFFEN J. BOGUSKI MS. ALTSCHUL SF. HORII A. ANDO H. MIYOSHI Y. MIKI Y. NISHISHO I AND NAKAMURA Y. (1991). Identification of FAP locus genes from chromosome 5q21. *Science*, **253**, 661-665.

KNOWLES MA. ELDER PA. WILLIAMSON M. CAIRNS JP. SHAW ME AND LAW MG. (1994). Allelotype of human bladder cancer. *Cancer Res*., **54**, 531-538.

LASKO D. CAVENEE W AND NORDENSKJOLD M. (1991). Loss of constitutional heterozygosity in human cancer. *Annu. Rev. Genet*., **25**, 281-314.

LATIF F. FIVASH M. GLENN G. TORY K. ORCUTT ML. HAMPSCH K. DELISIO J. LERMAN M. COWAN J. BECKETT M AND WEICHSELBAUM R. (1992). Chromosome 3p deletions in head and neck carcinomas: statistical ascertainment of allelic loss. *Cancer Res*., **52**, 1451-1456.

LEE NK. YE Y-W. LI X. SCHWEITZER C AND NISEN PD. (1994). Allelic loss on chromosome 13 can precede histological changes in head and neck cancer. *Int. J. Oncol*., **5**, 205-210.

LI XH. LEE NK. YE YW. WABER PG. SCHWEITZER C. CHENG QC AND NISEN PD. (1994). Allelic loss at chromosomes 3p, 8p, 13q, and 17p associated with poor-prognosis in head and neck cancer. *J. Natl Cancer Inst*., **86**, 1524-1529.

LOUGHRAN O. EDINGTON KG. BERRY LJ. CLARKE LJ AND PARKINSON EK. (1994). Loss of heterozygosity of chromosome 9p21 is associated with the immortal phenotype of neoplastic human head and neck keratinocytes. *Cancer Res*., **54**, 5045-5049.

MAESTRO R. GASPAROTTO D. VUKOSAVLJEVIC T. BARZAN L. SULFARO S AND BOIOCCHI M. (1993). Three discreet regions of deletion at 3p in head and neck cancers. *Cancer Res*., **53**, 5775-5779.

MAO L. LEE DJ. TOCKMAN MS. EROZAN YS. ASKIN F AND SIDRANSKY D. (1994). Microsatellite alterations as clonal markers for the detection of human cancer. *Proc. Natl Acad. Sci. USA*, **91**, 9871-9875.

MERLO A. GABRIELSON E. ASKIN F AND SIDRANSKY D. (1994). Frequent loss of chromosome-9 in human primary nonsmall cell lung cancer. *Cancer Res*., **54**, 640-642.

MORITA R. ISHIKAWA J. TSUTSUMI M. HIKIJI K. TSUKADA Y. KAMIDONO S. MAEDA S AND NAKAMURA Y. (1991). Allelotype of renal cell carcinoma. *Cancer Res*., **51**, 820-823.

NAWROZ H. VANDERRIET P. HRUBAN RH. KOCH W. RUPPERT JM AND SIDRANSKY D. (1994). Allelotype of head and neck squamous cell carcinoma. *Cancer Res*., **54**, 1152-1155.

OWENS W. FIELD JK. HOWARD T AND STELL PM. (1992). Multiple cytogenetic aberrations in squamous cell carcinoma of the head and neck. *Eur. J. Cancer Oral Oncol*., **28B**, 17-22.

PETO R. PIKE MC. ARMITAGE PE. BRESLOW NE. COX DR. HOWARD SV. MANTEL N. McPHERSON K. PETO J AND SMITH PG. (1976). Design and analysis of randomised clinical trials requiring prolonged observation of each patient. *Br. J. Cancer*, **34**, 585-612.

REES M. LEIGH SEA. DELHANTY JDA AND JASS JR. (1989). Chromosome 5 allele loss in familial and sporadic colorectal adenomas. *Br. J. Cancer*, **59**, 361-365.

ROWLEY H. JONES AS AND FIELD JK. (1995). Chromosome 18: a possible site for a tumour suppressor gene deletion in squamous cell carcinoma of the head and neck. *Clin. Otolaryngol*., **20**, 266-271.

SATO T. TANIGAMI A. YAMAKAWA K. AKIYAMA F. KASUMI F. SAKAMOTO G AND NAKAMURA Y. (1990). Allelotype of breast cancer: cumulative allele losses promote tumour progression in primary breast cancer. *Cancer Res*., **50**, 7184-7189.

SATO T. SAITO H. MORITA R. KOI S. LEE JH AND NAKAMURA Y. (1991). Allelotype of human ovarian-cancer. *Cancer Res*., **51**, 5118-5122.

TSUCHIYA E. NAKAMURA Y. WENG SY. NAKAGAWA K. TSUCHIYA S. SUGAND H AND KITAGAWA T. (1992). Allelotype of non-small cell carcinoma: comparison between loss of heterozygosity in squamous cell carcinoma and adenocarcinoma. *Cancer Res*., **52**, 2478-2481.

UZAWA K, YOSHIDA H, SUSUKI H, TANZAWA H, SHIMAZAKI J, SEINO S AND SATO K. (1994). Abnormalities of the adenomatous polyposis coli gene in human oral squamous cell carcinoma. *Int. J. Cancer*, **58**, 814-817.

VAN DER RIET P, NAWROZ H, HRUBAN RH, CORIO R, TOKINO K, KOCH W AND SIDRANSKY D. (1994). Frequent loss of chromosome 9p21-22 early in head and neck cancer progression. *Cancer Res.*, **54**, 1156-1158.

VOGELSTEIN B, FEARON ER, KERN SE, HAMILTON SR, PREISINGER AC, NAKAMURA Y AND WHITE R. (1989). Allelotype of colorectal carcinomas. *Science*, **244**, 207-211.

YAMAGUCHI T, TOGUCHIDA J, YAMAMURO T, KOTOURA Y, TAKADA N, KAWAGUCHI N, KANEKO Y, NAKAMURA Y, SASAKI MS AND ISHIZAKI K. (1992). Allelotype analysis in osteosarcomas: frequent allele loss on 3q, 13q, 17p and 18q. *Cancer Res.*, **52**, 2419-2423.

YOO GH, XU HJ, BRENNAN JA, WESTRA W, HRUBAN RH, KOCH W, BENEDICT WF AND SIDRANSKY D. (1994). Infrequent inactivation of the retinoblastoma gene despite frequent loss of chromosome 13q in head and neck squamous cell carcinoma. *Cancer Res.*, **54**, 4603-4606.