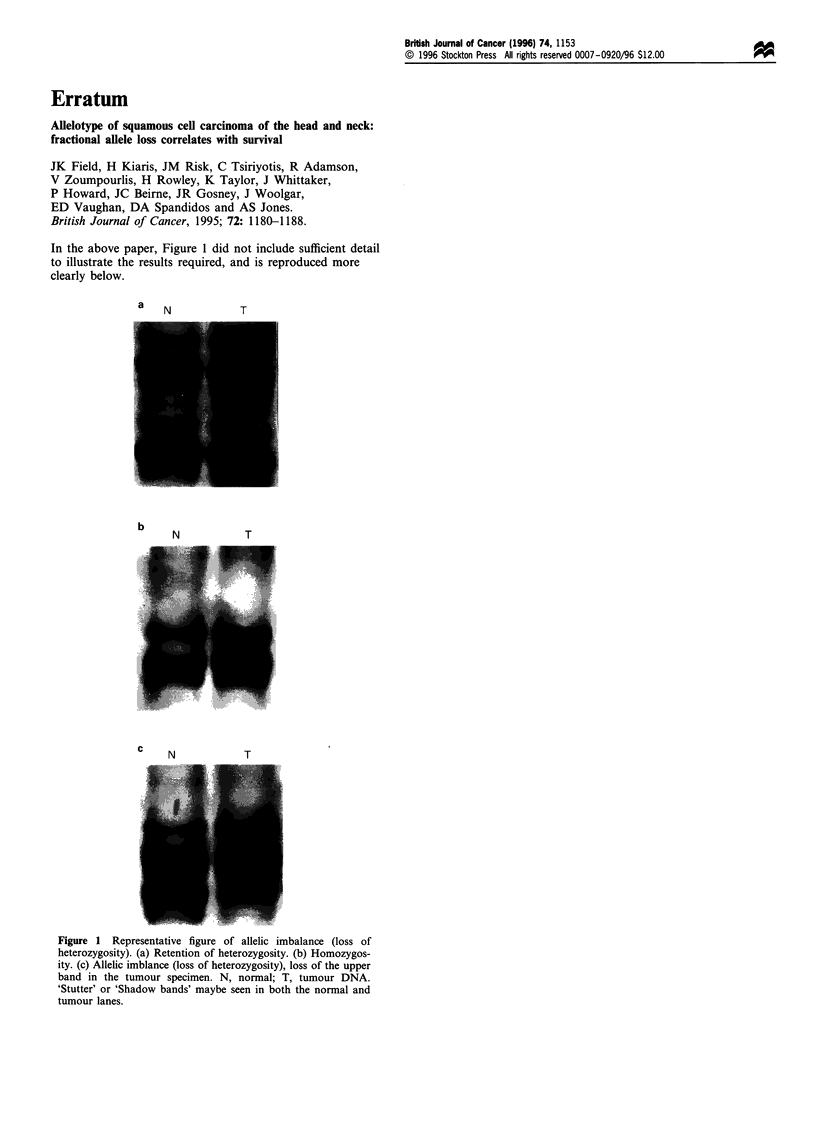# Allelotype of squamous cell carcinoma of the head and neck: fractional allele loss correlates with survival

**Published:** 1996-10

**Authors:** 

## Abstract

**Images:**


British Journal of Cancer (1996) 74, 1153
© 1996 Stockton Press All rights reserved 0007-0920/96 $12.00

### Erratum

**Allelotype of squamous cell carcinoma of the head and neck: fractional allele loss correlates with survival**

JK Field, H Kiaris, JM Risk, C Tsiriyotis, R Adamson, V Zoumpourlis, H Rowley, K Taylor, J Whittaker, P Howard, JC Beirne, JR Gosney, J Woolgar, ED Vaughan, DA Spandidos and AS Jones.
*British Journal of Cancer*, 1995; **72:** 1180–1188.

In the above paper, Figure 1 did not include sufficient detail to illustrate the results required, and is reproduced more clearly below.

**Figure 1** Representative figure of allelic imbalance (loss of heterozygosity). (a) Retention of heterozygosity. (b) Homozygosity. (c) Allelic imblance (loss of heterozygosity), loss of the upper band in the tumour specimen. N, normal; T, tumour DNA. 'Stutter' or 'Shadow bands' maybe seen in both the normal and tumour lanes.